# *Schistosoma mansoni* venom allergen-like proteins: phylogenetic relationships, stage-specific transcription and tissue localization as predictors of immunological cross-reactivity

**DOI:** 10.1016/j.ijpara.2019.03.003

**Published:** 2019-07

**Authors:** Leonardo P. Farias, Iain W. Chalmers, Samirah Perally, Henrique K. Rofatto, Colin J. Jackson, Martha Brown, Mariana I. Khouri, Mayra M.F. Barbosa, Paul J. Hensbergen, Cornelis H. Hokke, Luciana C.C. Leite, Karl F. Hoffmann

**Affiliations:** a Centro de Biotecnologia, Instituto Butantan, Av. Vital Brasil, 1500, 05503-900 São Paulo, SP, Brazil; b Instituto Gonçalo Moniz, Fundação Oswaldo Cruz (FIOCRUZ), Rua Waldemar Falcão, Salvador, Bahia, Brazil; c Institute of Biological, Environmental and Rural Sciences, Aberystwyth University, SY23 3FG Aberystwyth, UK; d Programa de Pós-Graduação Interunidades em Biotecnologia, Universidade de São Paulo, São Paulo, SP, Brazil; e Center for Proteomics and Metabolomics, Leiden University Medical Centre, P.O. Box 9600, 2300 RC Leiden, The Netherlands; f Department of Parasitology, Leiden University Medical Centre, P.O. Box 9600, 2300 RC Leiden, The Netherlands

**Keywords:** Venom allergen-like, Immunological cross reactivity, Tissue localization, *Schistosoma mansoni*

## Abstract

*Schistosoma mansoni* venom allergen-like proteins (SmVALs) are part of a diverse protein superfamily partitioned into two groups (group 1 and group 2). Phylogenetic analyses of group 1 SmVALs revealed that members could be segregated into subclades (A–D); these subclades share similar gene expression patterns across the parasite lifecycle and immunological cross-reactivity. Furthermore, whole-mount in situ hybridization demonstrated that the phylogenetically, transcriptionally and immunologically-related SmVAL4, 10, 18 and 19 (subclade C) were all localized to the pre-acetabular glands of immature cercariae. Our results suggest that SmVAL group 1 phylogenetic relationships, stage-specific transcriptional profiles and tissue localization are predictive of immunological cross-reactivity.

## Highlights

• The *Schistosoma mansoni* venom allergen-like (SmVAL) family relationships were investigated.
• Transcription patterns of SmVALs associate with phylogenetic relationships.
• There was clear antibody cross-reactivity between related native SmVAL proteins.
• SmVAL4, 10, 18 and 19 all localized via WISH to pre-acetabular glands of cercariae.

## Graphical abstract

**Figure:**
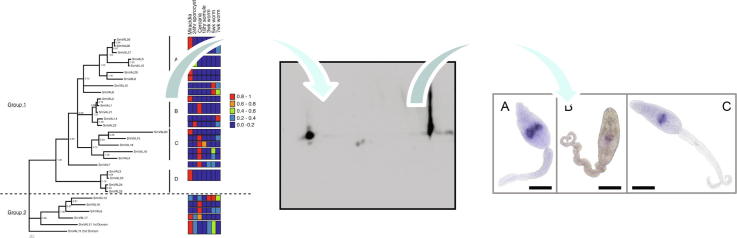

Schistosomiasis, a disease caused by parasitic flatworms within the genus *Schistosoma*, affects 200 million people in sub-Saharan Africa, South America and the Far East, with a further 600 million at risk of developing the disease (McManus et al., 2018). Currently, treatment against schistosomiasis relies on a single drug, Praziquantel (PZQ), which may lead to drug resistance and, over the long term, reduced efficacy (Doenhoff and Pica-Mattoccia, 2006). Therefore, the search for new sustainable control strategies and intervention targets remains an important priority.

In light of this objective, discoveries obtained through post-genomics investigation (Hokke et al., 2007) have led to studies focused on the identification of novel schistosome antigens putatively involved in the parasite/host interface. As a result, remarkable progress in the understanding of schistosome secretions and tegumental composition has been achieved, revealing the presence of multigenic protein families including the tegument allergen-like proteins (TALs), Tetraspanins, micro-exon gene (MEGs), Ly6 proteins and the venom-allergen like proteins (VALs) (reviewed in Wilson (2012)). While several members of these families are being examined for vaccine potential (e.g. *Schistosoma mansoni* tetraspanin 2 (SmTSP2); (Tran et al., 2006) and Sm29; (Cardoso et al., 2008)), an important aspect that has been overlooked is the extent to which immunological cross-reactivity occurs with respect to members of a given protein family.

Cross-reactive immune responses related to a group of proteins are crucially important in many areas of biology. However, the majority of research in this area focuses on inter-species/strain cross-reactivity, as for example the overlap in allergens between different foods (Kazatsky and Wood, 2016) and overlapping responses between species/strains of pathogens (Lee and Wilson, 2015, Cao et al., 2016). Nevertheless, there is an increasing understanding that immunological cross-reactivity occurring in response to similar protein family members in the same species can be critical to provide immunity against parasites. In malaria, a study of the vaccine candidate merozoite surface protein 3 (MSP3) showed that six related MSP3 genes presented conserved transcript expression profiles, similar localization and serological cross-reactivity (Singh et al., 2009). Limiting/avoiding cross-reactivity is also a key aspect in pathogen immune responses, in which multigenic families such as *P. falciparum* erythrocyte membrane protein 1 (PfEMP1) (Klein et al., 2014) and *Trypanosoma brucei* variant surface glycoproteins (VSG), are critical to pathogen survival (Cross, 1977). Importantly, in human schistosomiasis natural immunity has also been associated with cross-reactive antibody responses to related SmTAL proteins (Fitzsimmons et al., 2012). Given these findings, this study proposed to examine antigenic cross-reactivity within the *Schistosoma mansoni* VAL protein family, since a number of these members are present in parasite secretions and are also being investigated as anti-schistosomal vaccine candidates (Farias et al., 2011).

All SmVALs contain the SCP/TAPS (Sperm-coating protein/ Tpx-1/Ag5/PR-1/Sc7; Pfam PF00188) domain, which is a conserved α-β-α sandwich structure, ubiquitously found in family members across phyla. To date there have been 29 SCP/TAPS domain-containing proteins identified in *S. mansoni* (SmVAL1-28 from Chalmers et al. (2008) and SmVAL29 from Farias et al. (2012)). The SmVAL family is divided into two distinct sub-groups. All SmVALs from group 1 contain a predicted signal sequence and six highly conserved cysteine residues capable of forming disulphide bridges, indicating that these are secreted by the parasite. Group 2 SmVALs, which are fewer in number, consisting of only five members, are unlikely to be secreted due to their lack of a signal sequence as well as the six highly conserved cysteine residues. This is supported by the detection of multiple group 1 SmVALs in proteomic studies of parasite excretory/secretory (E/S) products including cercarial secretions (SmVAL4, 10 and 18; (Curwen et al., 2006, Hansell et al., 2008)), egg secretions (SmVAL2, 3, 5 and 9; (Cass et al., 2007)), miracidial/sporocyst secretions (SmVAL2, 3/23, 5/15, 9, 26/28, 27 and 29; (Wu et al., 2009, Farias et al., 2012)) and egg hatching fluid/secretions (SmVAL26/28; (Mathieson and Wilson, 2010, Farias et al., 2012)). Despite the abundance of group 1 SmVALs in these important life stages, the understanding of SmVAL function remains somewhat limited. A lipid-binding function was demonstrated for SmVAL4 (Kelleher et al., 2014), a plasminogen-binding function was revealed for SmVAL18 (Fernandes et al., 2017) and a host matrix metalloprotease modulatory function detected for SmVAL9 (Yoshino et al., 2014). The group 2 protein SmVAL6 has recently been shown to be a target for IgE, IgG4 and IgG1 responses in infected individuals, indicating that the SmVALs may influence the immune response (Farnell et al., 2015). However, these studies have limited their focus to individual SmVALs. Here, we demonstrate that phylogenetic, gene expression and spatial localization information can be used in combination to predict group 1 SmVAL immunological cross-reactivity. Further extending this analysis to the entire SmVAL family (and other multigenic families) may help assist in the selection of next generation of vaccine candidates.

The *S. mansoni* life cycle was maintained in hamsters or Tuck Ordinary (T.O.) mice (Harlan, USA) and experimental protocols were approved by both the Aberystwyth University (UK) animal welfare and ethical review body (AWERB, project license PPL 40/3700) and the Institutional Review Board on Animal Experimentation of the Butantan Institute, Brazil (CEUAIB; license no. 604-2009). *Schistosoma mansoni* cercariae, schistosomula, adult worms, eggs, miracidia and mother sporocysts (24 h and 96 h sporocysts) were obtained as previously described (Chalmers et al., 2008). Germ balls were obtained as previously described (Fernandes et al., 2017).

To examine whether antigenic cross-reactivity could occur between the SmVAL protein family members, we first compared the expression profiles of SmVALs at different stages of the parasite life cycle with respect to phylogenetic relatedness. Previous quantitative reverse transcription PCR (qRT-PCR) analysis provided gene expression quantification for 16 of the 29 *Smval* transcripts (1–13, 15, 21 and 23) (Chalmers et al., 2008). Herein we attempted to extend this analysis across the entire SmVAL family (data for SmVAL24 and 25 are absent due to amplification failures despite repeated attempts). Parasite materials, RNA extraction and cDNA synthesis as previously described (Chalmers et al., 2008) were used in qRT-PCR analyses of *Smvals 14, 16*–*20, 22* and *26*–*29* (qRT-PCR primers used can be found in Supplementary Table S1). In the cases of *Smval26, 27* and *28*, due to extremely high levels of sequence similarity (90–99% identity at the nucleotide level), a single combined expression profile representing these three transcripts was produced. This was also the case in a previous study for *Smval1/21, Smval3/23* and *Smval5/15* (Chalmers et al., 2008). Of the nine new expression profiles obtained, several displayed peak expressions in stages associated with parasite invasion or establishment in the mammalian host (e.g. *Smval14, 16, 18* and *19*), whilst others appear to be highly expressed in stages related to parasite invasion of the invertebrate host (e.g. *Smvals 20, 22, 26/27/28*, and *29*). The only exception was *Smval17*, which presented elevated expression in miracidia and cercariae stages (Supplementary Fig. S1).

The SmVAL expression profiles were then compared with SmVAL phylogenetic relationships to investigate whether closely related SmVALs (based on primary amino acid sequences) also shared common transcriptional patterns across the life cycle. To directly compare SmVAL expression profiles, data were converted to reflect relative abundance, in which the lifecycle stage with the highest expression value was designated as score 1.0, while expression in the other lifecycle stages was normalized accordingly. Expression data for *Smval1-13, 15, 21* and *23* was derived from data originally published by Chalmers et al. (2008). For phylogram construction, Bayesian phylogenetic analysis was performed as outlined in Chalmers et al. (2008) using an amino acid sequence alignment of the 30 SCP/TAPS domains with non-conserved regions removed. The phylogram showed four well-supported subclades (A–D) within the group 1 SmVALs (Fig. 1). An examination of qRT-PCR expression within these subclades revealed clusters of genes with very similar expression profiles, which is reflective of high sequence relatedness. For example, in subclade A, SmVAL5, 9, 15, 26, 27, 28 and 29 group together (posterior probability support value 1.00), all exhibiting high levels of expression within the miracidia life stage, yet minimal expression levels in most other life stages (Fig. 1). In subclade C, SmVAL4, SmVAL10, SmVAL18, SmVAL19 and SmVAL20 all group together (posterior probability support value 0.72) with all but SmVAL20 sharing a similar expression profile that peaks in the cercaria stage (Fig. 1). In contrast, subclade B presents a mixed profile, with one member (SmVAL2) showing peak expression in the miracidia stage, versus another (SmVAL22) in sporocysts, while others (SmVAL1 and 21) peak in cercariae and one (SmVAL14) shows elevated expression in the adult stage (Fig. 1). It is interesting to note that most group 1 proteins exhibit peak expression in the penetrating larval stages (miracidia and cercariae). The exceptions to this are SmVAL7, 8 and 12, which did not segregate into a distinct subclade, as well as the subclade B member SmVAL14.

**Fig. 1:**
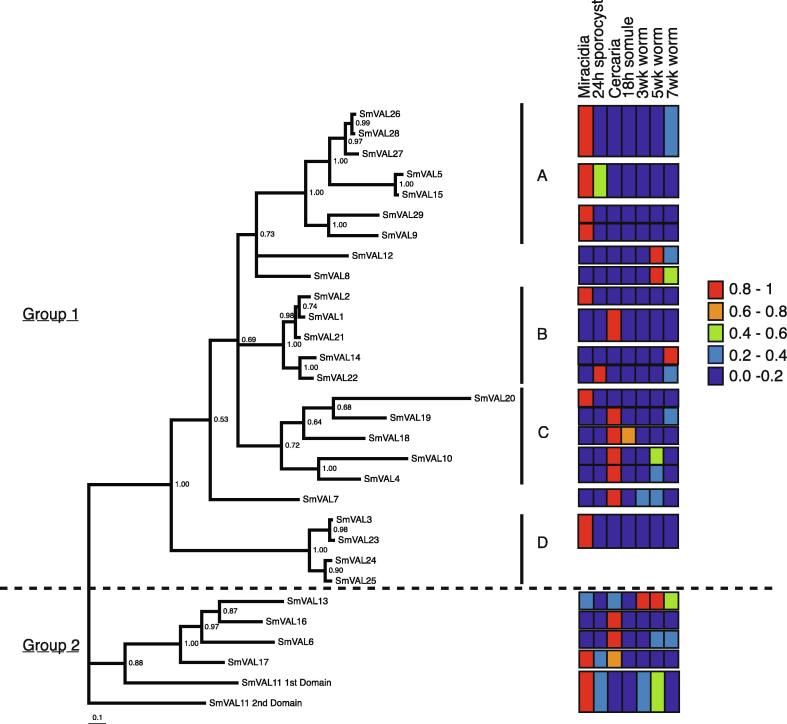
*Schistosoma mansoni* Venom allergen-like proteins (SmVAL) subclade divisions and expression profile across the parasite life cycle. Rooted phylogram illustration detailing the 30 SmVAL SCP/TAPS (Sperm-coating protein/ Tpx-1/Ag5/PR-1/Sc7) domain-containing proteins and distinctive subclades (A–D), accompanied by a heat map showing transcription levels (subject to data availability) at different stages determined by quantitative reverse transcription (qRT)-PCR analysis. Where highly similar transcripts could not be analysed separately by qRT-PCR, the data is represented jointly. qRT-PCR results for each *Smval* were transformed into proportional data, where the value of the life cycle with the highest expression for that *Smval* was set at 1 and all other life cycle transcript levels compared with that figure. Red represents any lifecycle stage between 0.8 and 1 compared with the highest measured transcript abundance, while orange (0.6–0.8), green (0.4–0.6), light blue (0.2–0.4) and dark blue (0–0.2) represent lower comparative expression levels. (For interpretation of the references to colour in this figure legend, the reader is referred to the web version of this article.)

Based on SmVAL sequence relatedness, phylogenetic relationships and expression profiles, we hypothesized that cross-reactivity would occur within the subclades, but not across different SmVAL family subclades. To investigate this, recombinant (r)SmVAL4 and 9 were produced in *Escherichia coli* as described in a previous study (Yoshino et al., 2014) and rSmVAL5 protein was generated in *Pichia pastoris* (Supplementary Fig. S2); antisera against each recombinant protein were raised in mice (Supplementary Data S1). These anti-sera were then used in western blotting experiments against recombinant proteins and native extracts to assess cross-reactivity as previously described (Rofatto et al., 2012). First, experimental confirmation that rSmVAL4 and rSmVAL5 were recognized by their respective antisera was obtained by one-dimensional SDS-PAGE of the recombinant proteins and Western blotting using anti-rSmVAL4 and anti-rSmVAL5 antisera (Fig. 2). While no cross-reactivity across subclades was detected, as anti-rSmVAL4 only recognized itself and anti-rSmVAL5 did not recognize rSmVAL4, there was evidence of cross-reactivity within the same subclade, with anti-rSmVAL5 antisera recognizing the SmVAL9 protein.

**Fig. 2:**
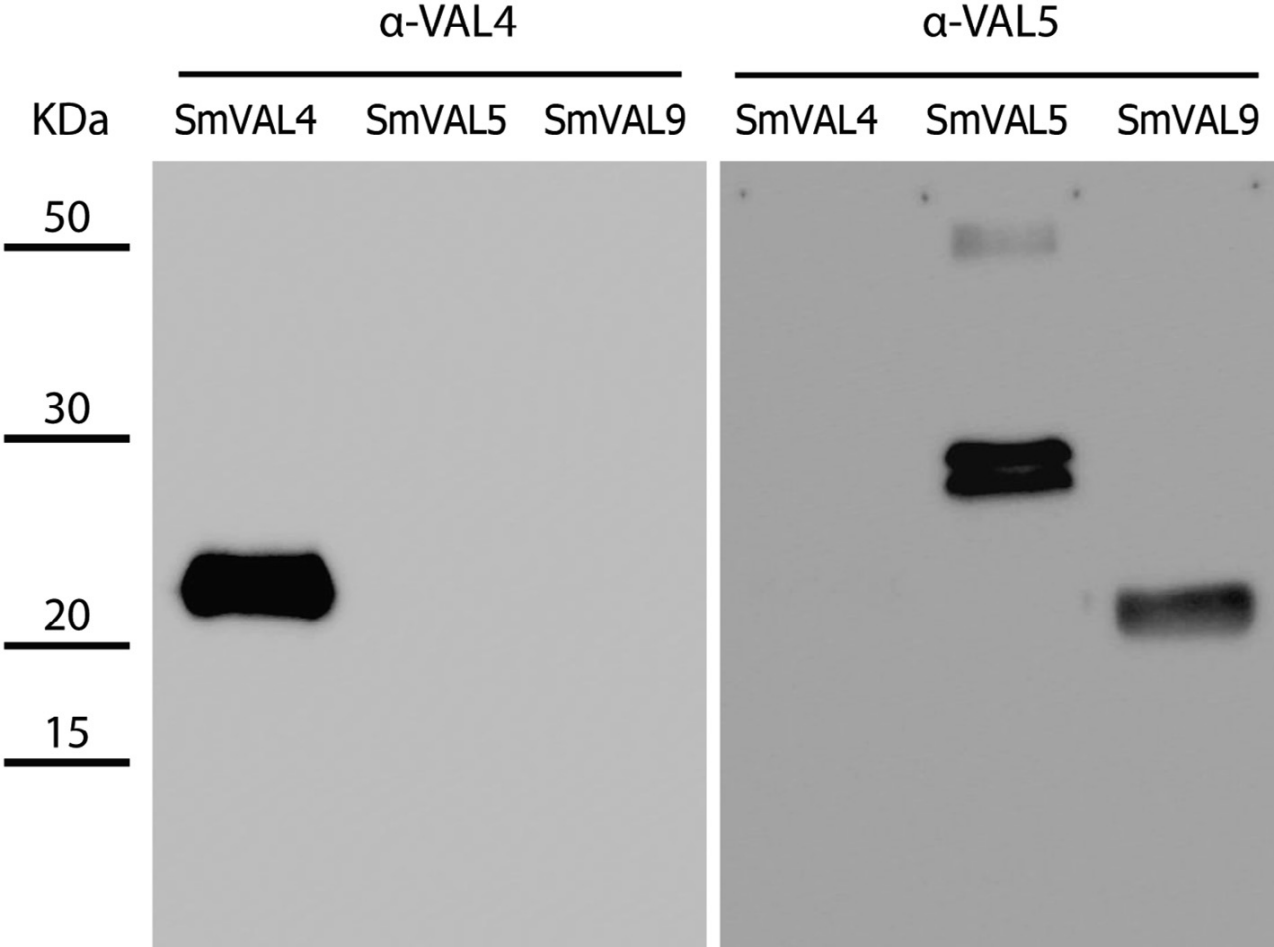
Anti-SmVAL (*Schistosoma mansoni* Venom allergen-like proteins) antisera cross-react only with intra-subclade members. Evaluation of cross-reactivity using recombinant SmVALs 4, 5 and 9 and anti-recombinant (r)SmVAL4 and anti-rSmVAL5 antisera. Recombinant proteins were submitted to western blotting (1.5 μg/lane). Blots were probed with murine antisera (1:15,000) from animals immunized with rSmVAL4 or rSmVAL5 expressed in *Pichia pastoris.* Signal was developed using a horseradish peroxidase (HRP)-conjugated anti-mouse IgG secondary antibody (Sigma no. A4416, 1:10,000) and enhanced chemiluminescence (ECL) prime reagent (GE healthcare no. RPN2232). Blots were exposed for 1 min and images were captured using a CCD camera (BioSpectrum Multi Imaging Unit, UVP, USA).

We then carried out two-dimensional gel electrophoresis (2-DE) Western blotting and mass spectrometric analysis to test for cross-reactivity within the A and C subclades by examining native SmVALs. Parasite protein extract preparations, electrophoresis, electroblotting, spot isolation, digestion and LC-MS/MS analysis are described in detail in Supplementary Data S1. For the subclade C analysis, proteins from cercariae secretions (0–3 h released products (RP)) were examined via 2-DE and blotted using anti-rSmVAL4 antisera. As predicted, due to the high sequence identity in this subclade (45–46%, complete protein sequence), anti-rSmVAL4 antisera recognized SmVAL4, SmVAL18 and SmVAL19 in the 0–3 h RP fraction (Fig. 3A, B, E and Supplementary Table S2). The results also showed two regions of cross-reactive signals (spots 2 and 5) that, upon LC-MS/MS analysis, yielded no significant hits to schistosome proteins (Fig. 3A and B). The predicted and observed molecular weight for some SmVALs differ significantly, likely due to protein glycosylation, as already demonstrated for SmVAL4, 10 and 18 (Jang-Lee et al., 2007, Farias et al., 2012). It is noteworthy that this is the first proteomics analysis reporting evidence of SmVAL19 protein detection in cercaria to schistosomula (0–3 h RP) secretions. Additionally, we observed a strong signal on the western blot (Fig. 3B, labeled as spot 5) in a pH region that resembles what was characterized as SmVAL10 protein by Curwen et al. (2006). However, we were unable to identify the SmVAL10 protein in our mass spectrometry analysis, likely due to low isoelectric focusing resolution.

**Fig. 3:**
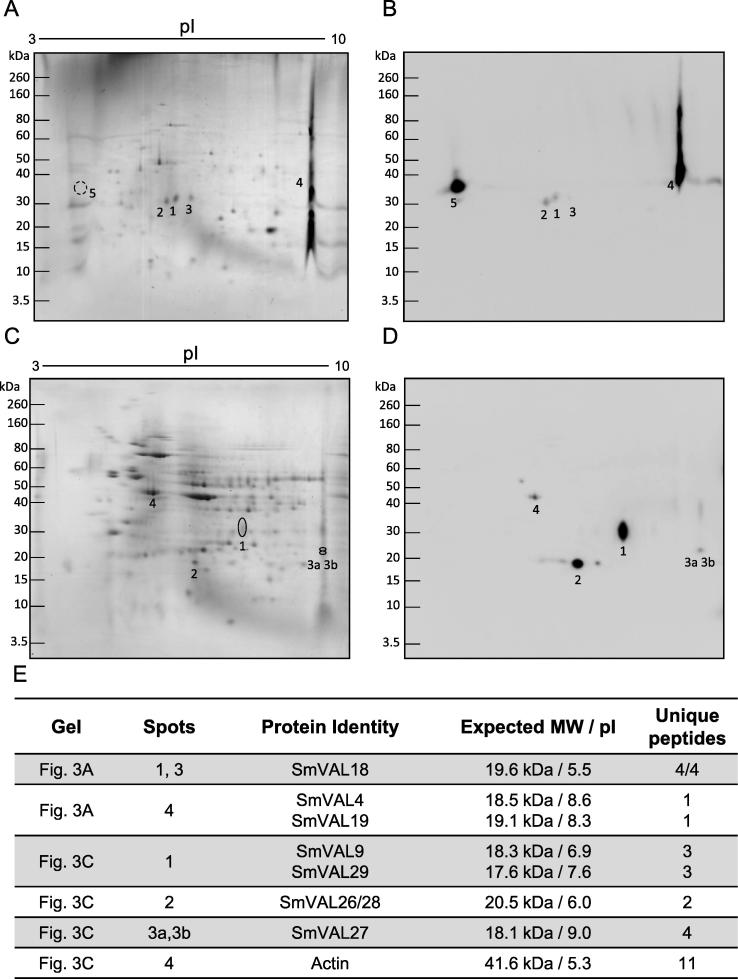
Intra-clade cross-reactivity of anti-rSmVAL4 (recombinant *Schistosoma mansoni* Venom allergen-like 4 protein) and anti-rSmVAL5 antisera against native SmVALs. (A) two-dimensional gel electrophoresis (2-DE) of 0–3 h cercarial/schistosomula released products (50 µg) stained with colloidal Coomassie. (B) Western blot of a replica gel presented in (A) revealing protein spots that cross-react with anti-rSmVAL4 antibodies. (C) 2-DE of soluble egg antigens (SEA) (56 µg) stained with colloidal Coomassie. (D) Western blot from a replica gel presented in (C) showing spots that cross-react with anti-rSmVAL5 antisera. Positions of molecular mass standards (kDa) are indicated on the sides of the gels. (E) Spots submitted to LC-MS/MS analysis and SmVAL protein identification. The predicted MW and average pI for each SmVAL identified as defined by Expasy (https://web.expasy.org/compute_pi/) and the number of unique peptides identified during mass spectrometry analysis are displayed. The full mass spectrometric analysis for all protein identities is detailed in Supplementary Tables S2 and S3.

To further evaluate cross-reactivity within subclade A, anti-rSmVAL5 antisera was applied to 2-DE blots of *S. mansoni* soluble egg antigen (SEA) preparations. SEA preparation, electrophoresis, electroblotting, spot isolation, digestion and LC-MS/MS analysis are described in detail in Supplementary Data S1. The anti-rSmVAL5 antisera recognized proteins with different levels of sequence identity, namely SmVAL9 (49%), SmVAL26/28 (62/63%), SmVAL27 (60%) and SmVAL29 (50%) (Fig. 3C, D, E and Supplementary Table S3). It should be noted that as SmVAL26 and SmVAL28 share almost identical primary sequences, neither of the peptides identified were discriminatory between these two SmVALs. Surprisingly, none of the proteins detected were identified as SmVAL5, again likely due to isoelectric focusing resolution or MS detection limitations.

To examine whether the subclade C members identified as cross-reactive shared the same localization within the parasite, we employed Whole-Mount In Situ Hybridization (WISH), which we believe is a stringent technique capable of accurately mapping spatial gene expression in parasite tissue. Prior to conducting WISH assays, we first evaluated the specificity of the SmVAL4, 10 and 18 RNA probes against their corresponding cDNAs by modified reverse northern blotting. Briefly, template cDNAs were generated via the amplification of previously cloned genes using PCR (Chalmers et al., 2008). Approximately 50 ng of the PCR products were run on a 1% agarose gel; after documentation, the gel was submitted to capillary blotting on Hybond-N+ charged nylon membranes (GE Healthcare, USA), followed by depurination, denaturation and neutralization, all carried out according to the manufacturer’s recommendations. Blocking, probe hybridization and washing steps, as well as detection techniques, were performed under the same conditions described below for the WISH assays. Images were captured using an Image Quant LAS 4000 photodocumentation system (GE Healthcare, USA). Our analysis revealed that, contrary to our results regarding the anti-rSmVAL4 antibody, the SmVAL RNA probes specifically detected their related cDNAs, with no evidence of cross-reactivity observed (Supplementary Fig. S3).

Next, WISH was used to determine the localization of *Smval4, 10 18* and *19* transcripts in immature cercariae. Additionally, *Smval1* localization (a subclade B protein expressed in the cercaria) was examined to ascertain whether different subclades possessed unique localizations within the parasite. The protocols used for fixation, permeabilization, in situ hybridization and staining of germ balls and immature cercariae were based on protocols previously described (Fernandes et al., 2017), and recently used to localize *Smval4* and *24* in the pre-acetabular glands of immature cercariae. Specific antisense RNA probes were synthesized with digoxigenin (DIG) in vitro using T7 or Sp6 RNA polymerase (Promega, Madison, USA) from cDNA sequences previously cloned in a pGEM-T easy vector (Supplementary Table S1). A *Smval4* DIG-labeled sense probe was used as a negative control. All five probes presented clear evidence of staining in the cercaria stage, represented by a band across the middle of the body along the anterior and lateral edges of the pre-acetabular glands (Fig. 4A–E). Confocal microscopy employing Alexa Fluor 647-conjugated lectin (PNA) and FITC-phalloidin was used to provide additional interpretation of the positioning of pre- and post-acetabular glands with musculature counterstaining (Fig. 4G and H), which revealed that two pairs of pre-acetabular glands lie anterior to the acetabulum, while three pairs of post-acetabular glands were located posterior to it. In addition, detectable levels of these transcripts were observed in other development stages of germ balls (Supplementary Fig. S4); in some of these, it was possible to view the two pairs of pre-acetabular glands as indicated by four blue spots (Supplementary Fig. S4C, G, H and L).

**Fig. 4:**
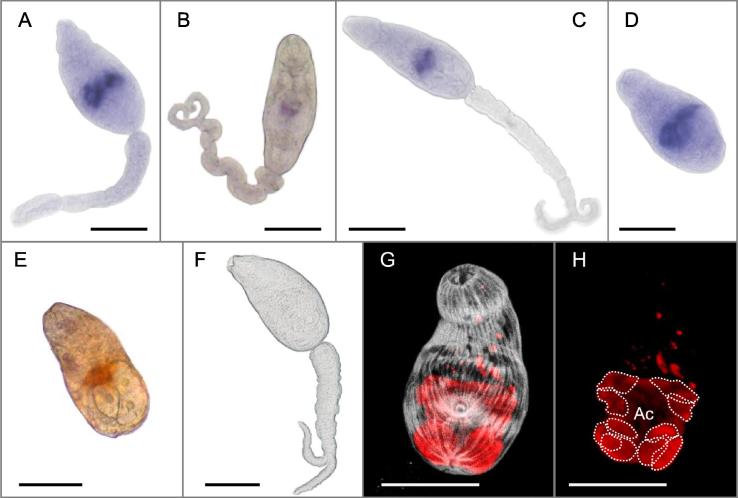
Localization of *Schistosoma mansoni* Venom allergen-like protein (SmVAL) transcripts to the pre-acetabular glands in *S. mansoni* cercariae. Whole mount in situ hybridization for *Smval1, 4, 10 18* and *19* transcripts in immature cercariae. (A–E) focal expression associated with the pre-acetabular glands of immature cercariae hybridized with *Smval1, 4, 10, 18* and *19* probes, respectively. (F) Negative control hybridized with a sense probe of SmVAL4. (G and H) A phalloidin-FITC stained parasite incubated with PNA-647 in the same orientation to aid interpretation. Note the acetabulum (Ac) and the two and three pairs of pre- and post-acetabular glands, respectively. Scale bars = 50 µm.

Recently, transcriptomic, microarray and proteomic studies have demonstrated the potential of SmVALs as prospective targets for immune intervention (reviewed in Chalmers and Hoffmann (2012)). The present study attempted to extend previous analyses of the transcriptional expression profiles of the SmVAL family (*Smval14-29*, with the exception of *Smval15, 21, 23 and 24/25*). Again, we broadly distinguished gene products that were associated with invasion or establishment in the mammalian host from others that appeared to be highly expressed in parasite stages related to the invasion of the invertebrate host.

In order to select an appropriate vaccine candidate in the context of a multi-gene family, it is crucial to consider cross-reactivity among members. Ultimately, cross-reactivity will determine the extent by which different antigens are recognized by the host immune system, which has obvious implications in vaccine design. In the case of the schistosome, immunization with antigens sharing epitopes (i.e. cross-reacting) with egg antigens presents a potential risk, since this may sensitize to egg proteins, thereby inducing an immune response against disintegrating eggs that could consequently exacerbate the pathological process. We have previously demonstrated the absence of antibody cross-reactivity between anti-rSmVAL6 (a group 2 protein) and recombinants SmVAL4, 7 and 26 (all group 1 proteins), which crucially have low sequence similarity to SmVAL6 (Rofatto et al., 2012). Data presented here extends these findings, indicating the absence of cross-reactivity among different subclades of the group 1 SmVALs, while demonstrating cross-reactivity within these subclades, such as in subclade C (between SmVAL4, 18 and 19) and in subclade A (between SmVAL5, 9, 26/28, 27 and 29). Moreover, the detection of SmVAL19 in cercaria secretions further expands the repertoire of SmVALs that are likely involved in host invasion.

Within clade cross-reactivity may not occur at low sequence identity levels though, as previous experiments using anti-rSmVAL4 failed to detect a cross-reactive protein (such as the clade C protein SmVAL20) in egg or miracidia extracts using 1D Western Blot experiments (Farias et al., 2012). In addition, due to the use of recombinant proteins to raise the anti-sera for these experiments, we cannot exclude the possibility that additional/different cross-reactivity could be detected using proteins with native folding and post-translational modifications. Therefore, a complete understanding of schistosome protein cross-reactivity may only be achieved with the use of anti-sera raised against native proteins due to schistosome-specific glycosylation such as was detected for SmVAL9 (Yoshino et al., 2014).

Our results demonstrate that anti-SmVAL antibodies cross-react with members from the same phylogenetic subclade (i.e. those sharing at least 30% sequence identity), suggesting that cellular localization studies using these anti-SmVALs antibodies could lead to misinterpretations. To circumvent this limitation, we employed the WISH technique, which is more stringent than immunohistochemistry due to the high probe hybridization temperatures used (60–65 °C). The WISH assays determined the tissue localization of *Smval1, 4, 10, 18 and 19* transcripts in the pre-acetabular glands of stubby-tailed, young elongating-tail and immature cercariae. These data corroborate and extend previous DNA microarray and proteomics data (Curwen et al., 2006, Chalmers et al., 2008, Hansell et al., 2008, Parker-Manuel et al., 2011), which imply that these SmVAL proteins may play a role in the early stages of infection. In light of the identification of SjVAL1 in the head gland of cercariae (Chen et al., 2010), it is tempting to speculate that some *S. mansoni* orthologs could also be localized in this structure. Our data, however, exclude this possibility, at least for SmVAL1, 4, 10, 18 and 19. In addition, SjVAL1 localization data was obtained using polyclonal antibodies, and as demonstrated here, multi-gene family immunolocalization data should be interpreted carefully due to cross-reactivity issues.

The detection of SmVAL1, 4, 10, 18 and 19 provided here, SmVAL4 and 24/25 described by Fernandes et al. (2017) and, potentially, SmVAL21, 22 (Parker-Manuel et al., 2011), in the acetabular glands indicates that 37.5% of all group 1 SmVAL proteins are expressed in this tissue. Although the amount of SmVAL4, 10 and 18 based on spot volume (∼3%) does not rival the five cercarial elastases (∼34.4% of the total spot volume) (Wilson, 2012), the large number of group 1 SmVALs (nine) potentially expressed in the acetabular glands calls attention to this location as a hotspot for this gene family. In conclusion, our results highlight the importance of establishing localization status, as well as determining the extent of cross-reactivity across the SmVAL family members in order to better define their vaccine potential.

## Appendix A Supplementary data

Supplementary data 2

Supplementary Fig. S1 *Schistosoma mansoni* Venom allergen-like (*Smval*) family transcription throughout the schistosome life cycle. Total RNA from indicated life stages was obtained and utilized for reverse transcription quantitative PCR (qRT-PCR) analysis to determine *Smval14-29* transcript abundance. For each *Smval* transcript, a bar graph is displayed indicating relative abundance (compared with alpha tubulin, *Smat1*) throughout the *S. mansoni* lifecycle. The X-axis indicates each specific life stage cDNA being tested. The Y-axis represents the ratio of SmVAL gene expression relative to that of *Smat1* (reference gene). Data are presented as mean ratios (+/− S.D.) from technical duplicates.

Supplementary Fig. S2 Expression and purification of recombinant *Schistosoma mansoni* Venom allergen-like 5 protein (rSmVAL5). (A) SDS-PAGE gel stained with coomassie, lanes 1 and 2, supernatant and inclusion bodies after lysis of the yeast, respectively. (B) The same gel stained with InVision (Invitrogen) for visualization of His-tagged fusion protein bands. (C) Lanes 1 and 2, solubilization of inclusion bodies with 8 M urea and 6 M guanidine, respectively. (D) The same gel stained with InVision for visualization of His-tagged fusion protein bands. (E) SDS-PAGE analysis of fractions from Ni^2+^-charged column chromatography; Lane 1, sample applied to the column, lanes 2-4, fractions containing the main peak of rSmVAL5 eluted by linear gradient of imidazole (20–500 mM).

Supplementary Fig. S3 Evaluation of SmVAL 4, 10 and 18 probe specificities by reverse northern blot. (A) Agarose gel (1%) containing approximately 50 ng of SmVALs 4, 10 and 18 cDNA (PCR products). (B) Nylon membranes hybridized with RNA probes for SmVALs 4, 10 and 18.

Supplementary Fig. S4 Transcript localization of *Smvals* 1, 10 and 18 in the pre-acetabular glands of germ balls and immature cercariae by Whole in-situ hybridization (WISH). (A) No clear staining was detected in young round germ balls. Transcript expression was identified in the pre-acetabular glands of stubby-tailed developing germ balls (B, G, H and L), as well as in the young elongating-tail stage (C, D, I, J, M, N and O) and in germ balls nearing maturity (E, K and P). (F) Negative control hybridized with sense probe of SmVAL4. Scale bars = 50 μm.
