# Supplementary material for: Schistosoma mansoni venom allergen-like proteins: phylogenetic relationships, stage-specific transcription and tissue localization as predictors of immunological cross-reactivity
Source: Int J Parasitol. 2019 Jul;49(8):593–9. doi: 10.1016/j.ijpara.2019.03.003 (PMC6598858; doi:10.1016/j.ijpara.2019.03.003)
Supplement: Supplementary data 1 [file mmc1.doc]

**1. Supplementary materials and methods**

*1.1. Cloning, expression and purification of recombinant Schistosoma mansoni venom allergen-like (SmVAL) proteins*

The full-length open reading frames for SmVAL4, 5 and 9 was previously validated (Chalmers et al., 2008). These genes were amplified by PCR (oligonucleotides are listed on Supplementary Table S1) from parasite-derived cDNA (Chalmers et al., 2008) with the predicted native signal peptide removed, and then cloned into the pGEM-T Easy vector system (Promega, USA]). Sub-cloning, expression and purification conditions of SmVAL9 have been previously described (Yoshino et al., 2014) with SmVAL4 expression following the same procedures. The cDNA fragment corresponding to SmVAL5 (from M49 to R243) was cloned into the pPICZα-A vector (Invitrogen, USA), followed by the transformation in *Pichia pastoris*; the screening of clones and protein expression were as previously described (Farias et al., 2012). As this protein was expressed intracellularly, cells were harvested and resuspended in 200 ml of lysis buffer (100 mM Tris pH 6.4, 0.3 M NaCl), then disrupted with a bead beater (Biospec model-11079000) for 10 cycles of 1 min each (with a 1 min interval between each cycle). The crude homogenate was centrifuged at 20,000 *g* for 40 min. For protein purification, the inclusion bodies of recombinant SmVAL5 (rSmVAL5) were rinsed twice with washing buffer (lysis buffer, 2% Triton X-100, 2 M urea), and then resuspended in solubilization buffer (lysis buffer plus 5 mM beta-mercaptoethanol, 20 mM imidazole, 6 M guanidine hydrochloride). The recombinant protein was then purified by metal affinity chromatography using the Akta Prime system (GE Healthcare, USA) under denaturing conditions. Briefly, the sample was loaded onto a Ni2+-NTA column (5 ml bed volume) pre-equilibrated with the same buffer. The column was then washed with 10 bed volumes of equilibration buffer and eluted with 20-500 mM imidazole linear gradient. Fractions encompassing the main peak and the purity of the preparation were assessed by SDS-PAGE. The protein was then refolded by slow dialysis in PBS pH 7.4 (PBS) with 5% glycerol containing decreasing concentrations of guanidine (4, 3, 2, 1 M and no guanidine) (Supplementary Fig. S2).

*1.2. Antisera preparation*

Polyclonal mouse serum was produced against preparations of rSmVAL4, as previously described (Farias et al., 2012), and rSmVAL5, as described herein. Briefly, BALB/c mice were immunized four times at 7 day intervals with 10 µg of protein. For the initial dose, proteins were formulated with TiterMax Gold adjuvant (Sigma, USA) as per the manufacturer’s recommendations, while proteins were inoculated in PBS 1X for subsequent doses. Fifteen days after the final inoculation, the rodents were exsanguinated.

*1.3. Two-dimensional (2-D) PAGE of native schistosome proteins*

To obtain the proteins released by cercariae, mechanical transformation was performed by vortexing to stimulate the release of gland cell contents of ≈ 115,000 cercariae, which were then cultured for 3 h in 9 ml of RPMI 1640 medium (Invitrogen) at 37°C under 5% CO_2_. Parasites were collected by centrifugation at 200 *g* at 4° C for 5 min and the supernatant (medium containing released proteins), termed 0-3 h RP, was stored at -20 °C with the addition of a protease inhibitor (Roche Diagnostics, Mannheim, Germany). Soluble protein extracts from eggs (SEA) were prepared in 40 mM Tris, pH 7.4, plus protease inhibitor (Roche Diagnostics, Germany) by sonication on ice (four cycles of 50 s, 40% amplitude, with 2 min intervals between each cycle). Samples were centrifuged at 17,000 g for 30 min at 4°C and the supernatant was recovered and stored at -20 °C. Protein concentrations were determined with a Bradford Assay Kit (Sigma-Aldrich, USA).

For 2-D polyacrylamide gel electrophoresis (2-DE), 0-3 h RP preparations were precipitated by adding 0.25 volume of 100% trichloroacetic acid to samples followed by freezing at -20°C for 1 h. Proteins were pelleted after centrifugation at 17,000 g for 20 min at 4°C. The protein pellet was washed twice in acetone in an ice bath. Once the protein pellet was air-dried, it was resuspended in isoelectric focusing (IEF) buffer (C1) and quantified by a BCA protein assay.

A total of 50 µg or 250 µg of protein sample was used to passively rehydrate and focus 7 cm pH 3-10 non-linear IPG strips (Biorad, UK) at 20°C for protein separation in the first dimension. IPG strips were focused between 10000-15000 Vh using a Biorad IEF focusing machine. Each IPG strip was equilibrated for 15 min in 3 ml of equilibration buffer (50 mM Tris-HCl, pH 8.8, 6 M urea, 30% v/v glycerol and 2% w/v SDS) with the addition of 20 mg/ml of dithiothreitol (DTT). The equilibration buffer containing DTT was removed, and IPG strips were then incubated for another 15 min in the same buffer with iodoacetimide (IAA) at 25 mg/ml. The focused proteins were separated in the second dimension on NuPage Novex pre-cast 4-12% Bis-Tris gradient gels (Invitrogen, USA) at 200 V for 35 min using the XCell SureLock® Mini-Cell electrophoresis system (Invitrogen, USA). Gels were run in duplicate to provide a reference gel for protein spot identification by western blotting. Reference gels were stained with colloidal Coomassie and background staining was removed using 1% acetic acid. All Coomassie-stained gels were imaged with a GS-800 calibrated densitometer (Biorad, UK) set for Coomassie staining at 700 dpi. Key protein spots of interest were excised for in-gel tryptic digestion.

*1.4. Western blotting*

Resolved proteins on 1-D or 2-D PAGE gels were blotted onto polyvinyl difluoride (PVDF) membranes in NuPAGE transfer buffer (Invitrogen). Western blots were blocked in blocking buffer (50 mM Tris, pH 7.5, 0.3% Tween containing 5% skimmed milk powder) prior to incubation for 3 h with the primary antibody (1:3500 dilutions of anti-rSmVAL4, anti-rSmVAL5, or normal mouse serum) in antibody diluent (50 mM Tris, pH 7.5, 0.05% Tween, 5% skimmed milk powder and 150 mM NaCl). Blots were washed three times in 50 mM Tris-HCl, pH 7.5, before incubation for 1 h with secondary horseradish peroxidase-conjugated anti-mouse IgG antibody (Sigma) diluted 1:5000 in antibody diluent. After washing again in 50 mM Tris-HCl, pH 7.5, blots were developed using ECL-Plus reagent (GE Healthcare) and images were captured using a CCD camera (BioSpectrum Multi Imaging Unit, UVP, USA]).

*1.5.* *In-gel tryptic digestion*

Selected spots were cut from colloidal Coomassie-stained gels and stored in 10% ethanol/1% acetic acid at 4 °C until further use. For trypsinization, the gel plugs were cut into small pieces, and after two rounds of washing with 25 mM NH_4_HCO_3_ and dehydration with 100% acetonitrile, the gel particles were completely dried in a centrifugal vacuum concentrator (Eppendorf, Hamburg, Germany). Dried gel particles were re-swollen for 15 min on ice by addition of 15 µl of a trypsin solution (12.5 ng/µl in 25 mM NH_4_HCO_3_ sequencing grade modified trypsin, Promega, Madison, WI, USA). Subsequently, 20 µl of 25 mM NH_4_HCO_3_ were added and samples were kept on ice for an additional 30 min, followed by tryptic digestion overnight at 37 °C. The overlaying digestion solution was collected. Two additional rounds of extraction with 20 µl of 0.1% TFA were used to extract peptides from the gel plugs and the extracts were pooled.

*1.6. Nano LC ESI MS/MS*

Nanoflow LC was performed on an Ultimate LC system (Dionex, Sunnyvale, CA, USA). A volume of 10 µL of sample was injected onto a C18 PepMapTM 0.3 mm×5 mm trapping column (Dionex, USA) and washed with 100% A (2% acetonitrile in 0.1% formic acid in MQ water, v/v) at 20 µL/min for 15 min. Following valve switching, peptides were separated on a C18 PepMap 75 µm×150 mm column (Dionex) at a constant flow of 200 nL/min. The peptide elution gradient was from 10 to 60% B (95% acetonitrile in 0.1% formic acid in ultrapure water v/v) over 50 min. The nanoflow LC system was coupled to an HCTultra IonTrap (Bruker Daltonics, Bremen, Germany) using a nano-electrospray ionisation source. The spray voltage was set at 1.2 kV and the temperature of the heated capillary was set to 165 °C. Eluting peptides were analyzed using the data-dependent MS/MS mode over a 300–1500 m/z range. The five most abundant ions in an MS spectrum were selected for MS/MS analysis by collision-induced dissociation using helium as the collision gas.

*1.7. Mass spectrometry data analysis*

Peak lists were generated using DataAnalysis 4.0 software (Bruker Daltonics, USA) and exported as Mascot Generic (MGF) files. These files were searched against the *S. mansoni* predicted protein sequences database (v4.0h, www.genedb.org) using the Mascot (version 2.2.1) search algorithm (Matrix Science, London, UK) and data from one plug were merged using Mascot Deamon. A peptide mass tolerance of 0.6 Da (with # 13C = 1) and a MS/MS fragment tolerance of 0.5 Da was used. Trypsin was designated as the enzyme and up to one missed cleavage site was allowed. Carbamidomethylcysteine was selected as a fixed modification and oxidation of methionine as a variable modification. Only significant protein hits with at least one unique peptide with a score above 30 were selected.

References

Chalmers, I.W., McArdle, A.J., Coulson, R.M., Wagner, M.A., Schmid, R., Hirai, H., Hoffmann, K.F., 2008. Developmentally regulated expression, alternative splicing and distinct sub-groupings in members of the *Schistosoma mansoni* venom allergen-like (SmVAL) gene family. BMC Genomics 9, 89.

Farias, L.P., Rodrigues, D., Cunna, V., Rofatto, H.K., Faquim-Mauro, E.L., Leite, L.C., 2012. *Schistosoma mansoni* venom allergen like proteins present differential allergic responses in a murine model of airway inflammation. PLoS Negl Trop Dis 6, e1510.

Yoshino, T.P., Brown, M., Wu, X.J., Jackson, C.J., Ocadiz-Ruiz, R., Chalmers, I.W., Kolb, M., Hokke, C.H., Hoffmann, K.F., 2014. Excreted/secreted *Schistosoma mansoni* venom allergen-like 9 (SmVAL9) modulates host extracellular matrix remodelling gene expression. Int J Parasitol 44, 551-563.
