# Supplementary material for: Schistosoma mansoni venom allergen-like proteins: phylogenetic relationships, stage-specific transcription and tissue localization as predictors of immunological cross-reactivity
Source: Int J Parasitol. 2019 Jul;49(8):593–9. doi: 10.1016/j.ijpara.2019.03.003 (PMC6598858; doi:10.1016/j.ijpara.2019.03.003)
Supplement: Supplementary data 2 [file mmc2.doc]

# Supplementary Table S1. DNA sequences used to clone *Schistosoma mansoni* venom allergen-like (SmVAL) cDNAs in pET30a (+) and pPICZα-A vectors, to quantify transcript abundance of SmVAL14-29 by quantitative reverse transcription PCR (qRT-PCR) and to detect gene SmVAL spatial expression by Whole in-situ Hybridisation (WISH).

| **pET30a (+)** | | | | | |
| --- | --- | --- | --- | --- | --- |
| SmVAL | Gene DB | | Forward primer (5’-3’) | Reverse Primer (5’-3’) | |
| 4 | Smp_002070 | | TCTAGAATGAaGttatcggaaggacaacgagc | | CTCGAGTTCTGATTTATTACATTTACTTTC |
| 9 | Smp_176180 | | TCTAGAATGAAAATGAATGACACGATTCGTG | | CTCGAGTGCAGTCCTATACGGTCTTTGTTC |
| **pPICZα-A** | | | | | |
| 5 | Smp_179480 | | acgaattcatggacaatgccaccagagagaag | | cttctagactttcgtttgtatgattacacct |
| **qRT-PCR** | | | | | |
| SmVAL | Gene DB | | Forward primer (5’-3’) | | Reverse Primer (5’-3’) |
| 14 | Smp_078490 | | CAACCATTAATTCAGCAGTGGA | | CCTGGACCATAGTAACACACGA |
| 16 | Smp_124070 | | GTCGTGAAGCAACTCGAAAT | | CCTGCTTTAATAGTTGACTTCC |
| 17 | Smp_163400 | | TGCACAAAAATATGCAGAACA | | CATCTGCGCCTGTAAAATCT |
| 18 | Smp_001890 | | CCAAAACAACCTCCAGCAAA | | CCACCGCTTTTTCAATTGTT |
| 19 | Smp_123090 | | TCATTGTTCGTTTGCACTTCA | | TCATTCCAACGCAATACTTCC |
| 20 | Smp_127130 | | CAGCGGATAATTCGGATGTAA | | ACAATGTCCAACTGAGCAACC |
| 22 | Smp_139450 | | CAAAATATGGCTTTTGCTTCAA | | ACTGTCTTTAGAAATATTTTGCAC |
| 26  27  28 | Smp_154260  Smp_154290  Smp_176160 | | TTCTGTCTGATCAATGTCGT | | CGGTTTTGATGTCTGTCGC |
| 29 | Smp_120670 | | ACTGGGCTCTTACACCTCGAT | | CTCCAGGACCGTAATTGCATA |
| **Templates for RNA probe synthesis** | | | | | |
| 1 | | aacggtggattagatgcgaaaagtgaagagttgctcaatctgcatagaaaatacagacaagatttagttgattgtaaagttgatggacaacctccagctaaatatatgtcaccattgaagtggaatcacgatttagctcgacaagcacaatcattggcaaacaaatgcatcttacgtcacgacaaacgacattcgaatcaattcagttgggttggacaaaatatagctctccatccaaccattaagtcaggagtggatgcttggttcaatgaacacaaattatacaattacaatacgaacaactgtcctcaatgtttgcattacacacaaatggcttgggccaagaccacagacattggatgtggagttgcgaattgtccacggtacggtctatcaatcgtgtgtaattacggtccagggggtaattggaataatgagaaaccatatgaaatgaaaccacgtaatttgtgcccaaaagtgcaaaatattcctaaaaacagtttacaaactaacagtgctcatacacaacatggaccgaagccattaacgcaaagcgaagaaagggtgtcaacgaacgttcaaaatggacgtagagaatgcaatcaacgcgaacagttgagaagtagatattaa | | | |
| 4 | | aagttatcggaaggacaacgagcaatctataatttccataaaaaagttcgtaaagatgtaaaaaattgcaggatacctggtcaacctccagctaaaaatctaacaaagttgaaatggaataaactattagctaataaagctaaacaacaagctaaacgatgtaaatatgattcaaatgatccaaatgattttattattggggattttgaatcaattggacaaaatttagccgattatccaacaattgaaggtgcaatgaaagattggttagaagagtataaaaattataattttgaaaagaatcaatgtaatggtgattgtaaaaattataaacagatggtttggaataccactgaagaaataggctgcggttatgaaaaatgtggaaagaactatttgattgtttgcaattatgcaccaggggactctgaagatagaccatatgaagctaaaccagaaagtaaatgtaataaatcagaataa | | | |
| 10 | | gataagtcatctacgaaagagttgattttcaattttcataataaaattcgtgaagatgtatttaaaggtgttttatctggtcaacctaaagcgaaaaagatgtccaaactgaaatggaataaacttttagctaaattagctaaaggtcacgttcagaaatgtattcttgatagtggagatcttggaaaattatacgttggtaaatttgattctgtcggacaaactgtagcagaacatacttcaatacaaaacatattagatacatggttagaagaaaaaaatgattacgatttagacaagaatacatgtgaaaatgaatgtggaaactataaacaattagtgtgggctaacacaacggatattggatgtgcttcaaataaatgtggtaacaggtatatggtcgtatgtaattacgctccaggggctgatgatgaaaggccatatgaaaaagactcataa | | | |
| 18 | | ccaaaacaacctccagcaaaatatatgtcaaaattacaatggaataaacatttagcagaaaaagctcaactcacagctagccgatgtgattactcttatgatagtccaagtgatatgcgttttgaagagttttcttcagtggcacaaaatattgctgatagtccaacaattgaaaaagcggtggccagttggtttattgagtacaagaactattcttttgatgataacacatgcaaagatacatgtatgcagtataaacagatggtaaaaggtgaagaaactgaaattggttgtggtgtacaaaaatgttcaaacagatttctagtagtatgtaattattcaccagcagctgaagaagataaacaaccgtatgaaaaaggtactcaagaaaattgcgatgatgttgatgatgcagaatattaa | | | |
| 19 | | ctcgagatgaataagaatcatcccttgcattttgttatcttttcattgttcgtttgcacttcaaatggtgctaagaagaatctaacacaaaaattagaaactatacatcgtttacatacatattatagaaattctattctactttgtaaagttccaacacaacctccagctgaagatatggaagtattgcgttggaatgatattttagccaataatgctcaacaagtagcaaataaatgtgatttaaattttgatttagtcaatgataaactactggaacattttgaatcgattggacaaaatgtagctgaatcggatacaattaaaaatgcaatggaaaattggttccgagaatatcataattat | | | |

Restriction sites are underlined: *Xba*I (forward) and *Xho*I (reverse) for SmVAL4 and SmVAL9, and *EcoR*I (forward) and *Xba*I (reverse) for SmVAL5.
