# Supplementary figures and images for: Schistosoma mansoni venom allergen-like proteins: phylogenetic relationships, stage-specific transcription and tissue localization as predictors of immunological cross-reactivity
Source: Int J Parasitol. 2019 Jul;49(8):593–9. doi: 10.1016/j.ijpara.2019.03.003 (PMC6598858; doi:10.1016/j.ijpara.2019.03.003)

Gene expression relative to SmAT1

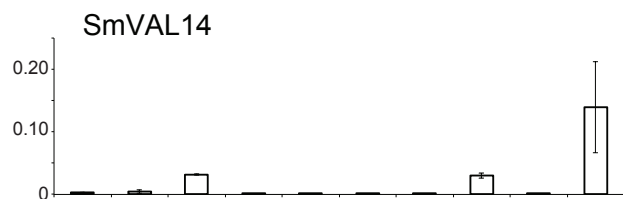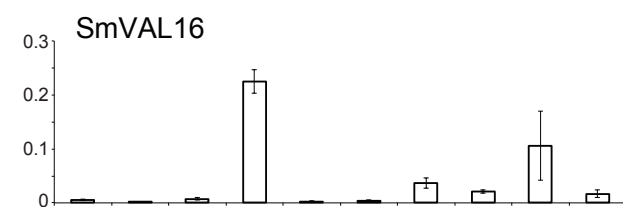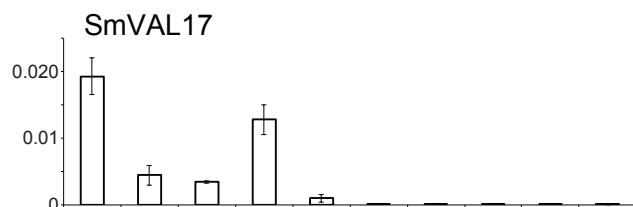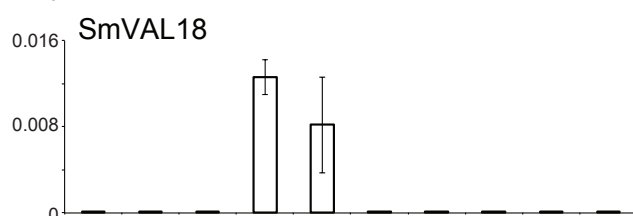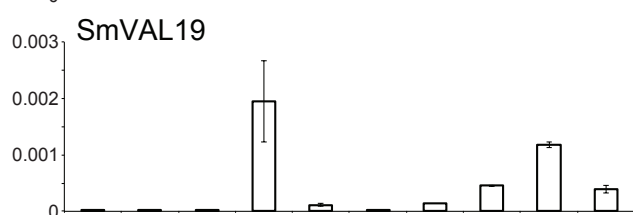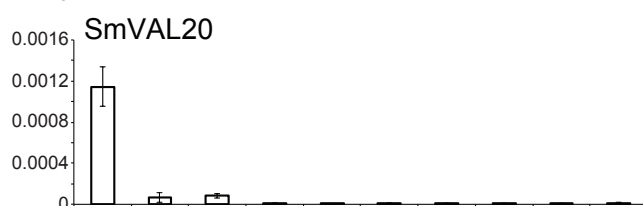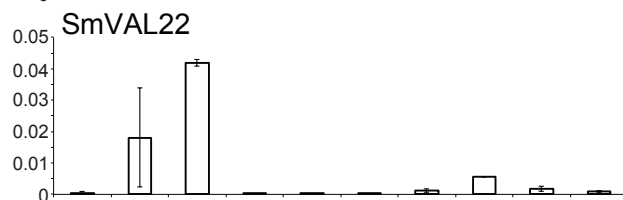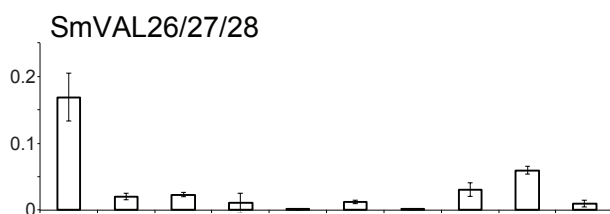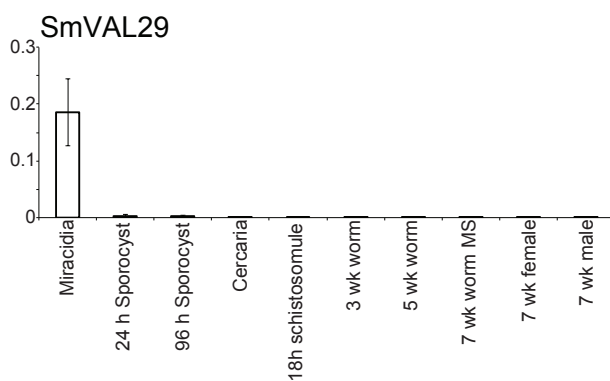

Supplement: Supplementary Fig. S1 — Schistosoma mansoni Venom allergen-like (Smval) family transcription throughout the schistosome life cycle. Total RNA from indicated life stages was obtained and utilized for reverse transcription quantitative PCR (qRT-PCR) analysis to determine Smval14-29 transcript abundance. For each Smval transcript, a bar graph is displayed indicating relative abundance (compared with alpha tubulin, Smat1) throughout the S. mansoni lifecycle. The X-axis indicates each specific life stage cDNA being tested. The Y-axis represents the ratio of SmVAL gene expression relative to that of Smat1 (reference gene). Data are presented as mean ratios (+/− S.D.) from technical duplicates. [file mmc3.pdf]

## Slide 1
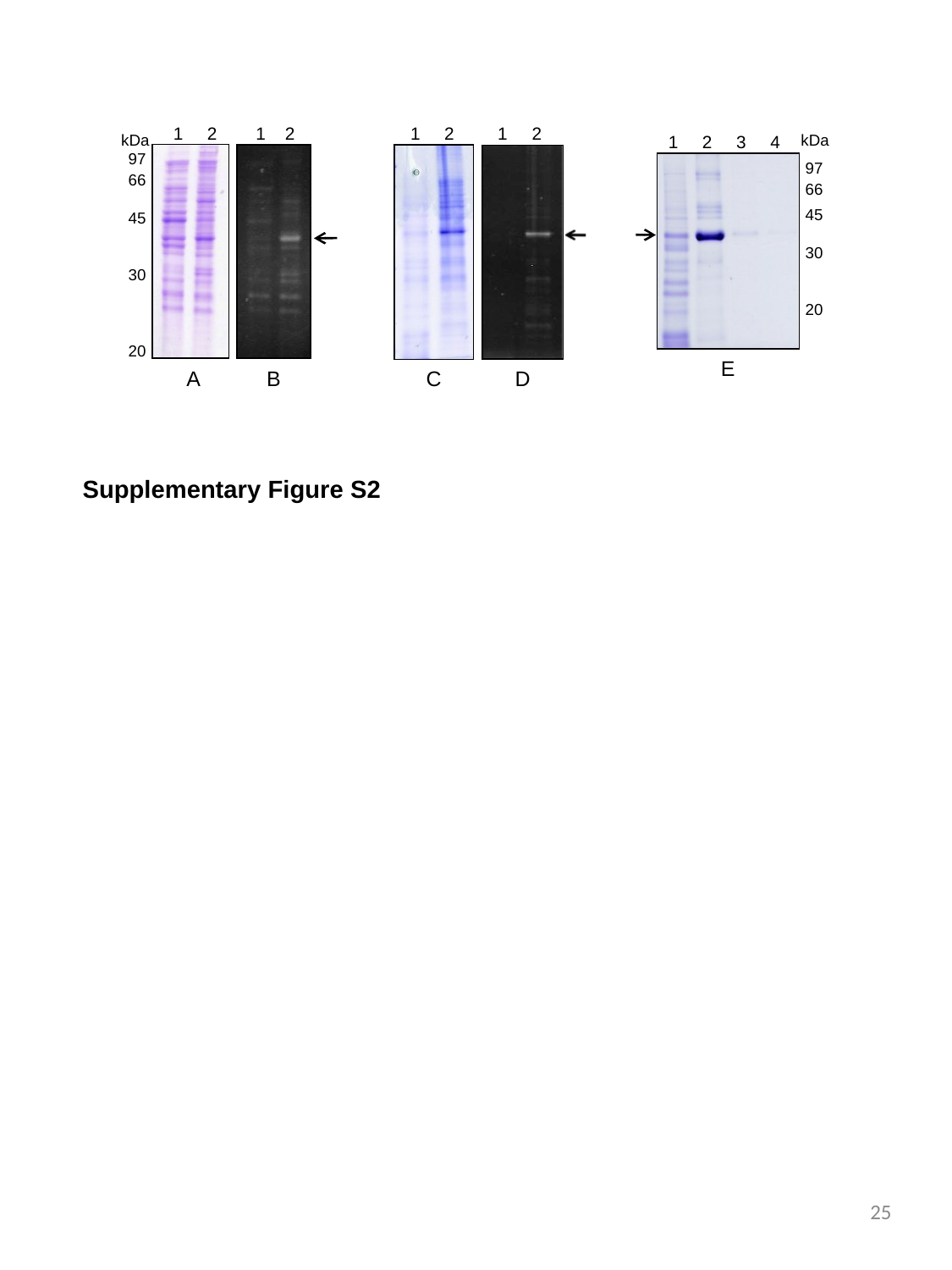

1 2 1 2
C
D
 1 2 1 2
97
66
45
30
20
B
 A
kDa
 1 2 3 4
97
66
45
30
20
E
kDa
Supplementary Figure S2
25

Supplement: Supplementary Fig. S2 — Expression and purification of recombinant Schistosoma mansoni Venom allergen-like 5 protein (rSmVAL5). (A) SDS-PAGE gel stained with coomassie, lanes 1 and 2, supernatant and inclusion bodies after lysis of the yeast, respectively. (B) The same gel stained with InVision (Invitrogen) for visualization of His-tagged fusion protein bands. (C) Lanes 1 and 2, solubilization of inclusion bodies with 8 M urea and 6 M guanidine, respectively. (D) The same gel stained with InVision for visualization of His-tagged fusion protein bands. (E) SDS-PAGE analysis of fractions from Ni2+-charged column chromatography; Lane 1, sample applied to the column, lanes 2-4, fractions containing the main peak of rSmVAL5 eluted by linear gradient of imidazole (20–500 mM). [file mmc4.pptx]

## Slide 1
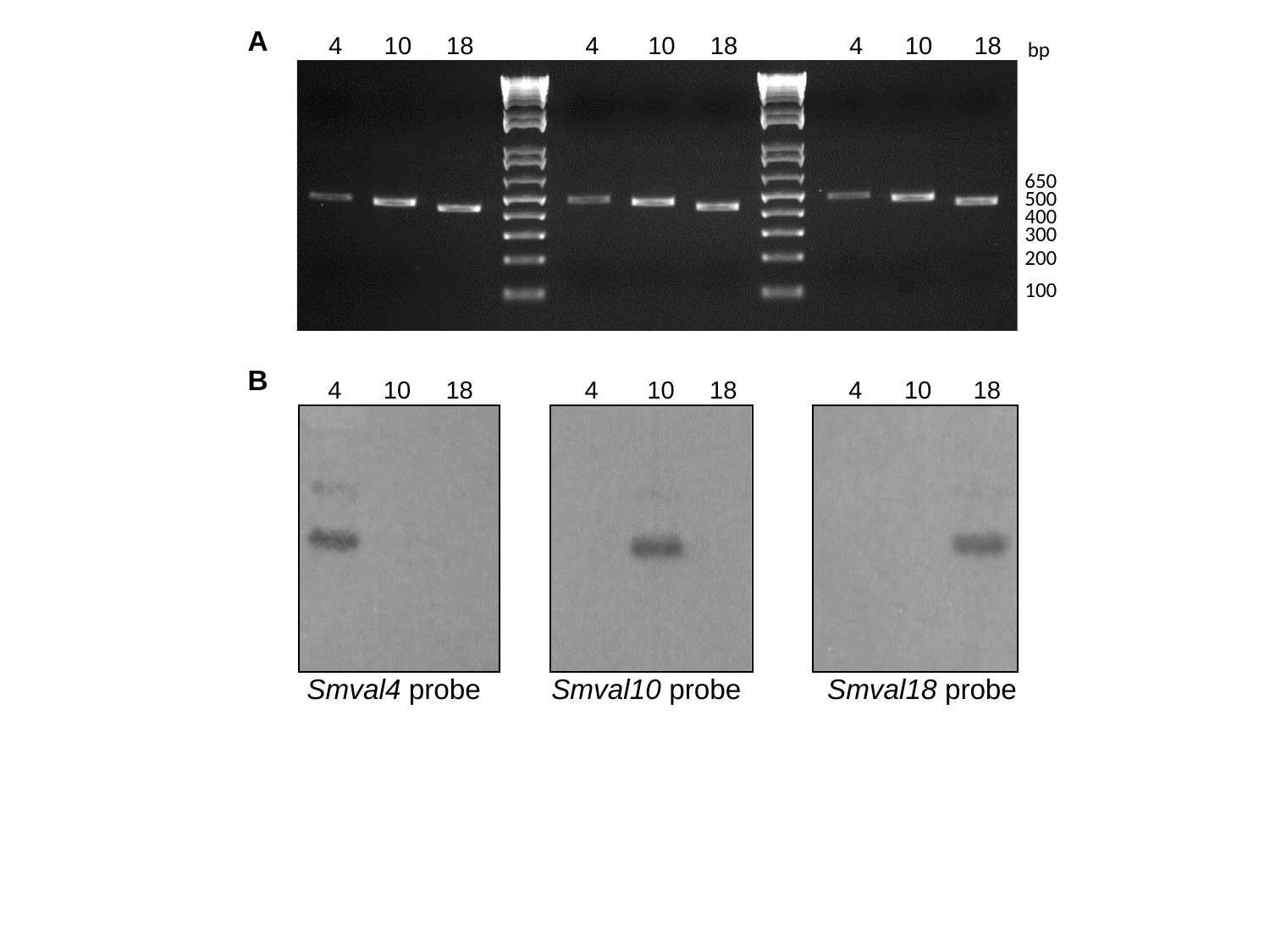

A
 4 10 18 4 10 18 4 10 18
B
 4 10 18 4 10 18 4 10 18
Smval4 probe Smval10 probe Smval18 probe
650
500
400
300
200
100
bp

Supplement: Supplementary Fig. S3 — Evaluation of SmVAL 4, 10 and 18 probe specificities by reverse northern blot. (A) Agarose gel (1%) containing approximately 50 ng of SmVALs 4, 10 and 18 cDNA (PCR products). (B) Nylon membranes hybridized with RNA probes for SmVALs 4, 10 and 18. [file mmc5.ppt]

## Slide 1
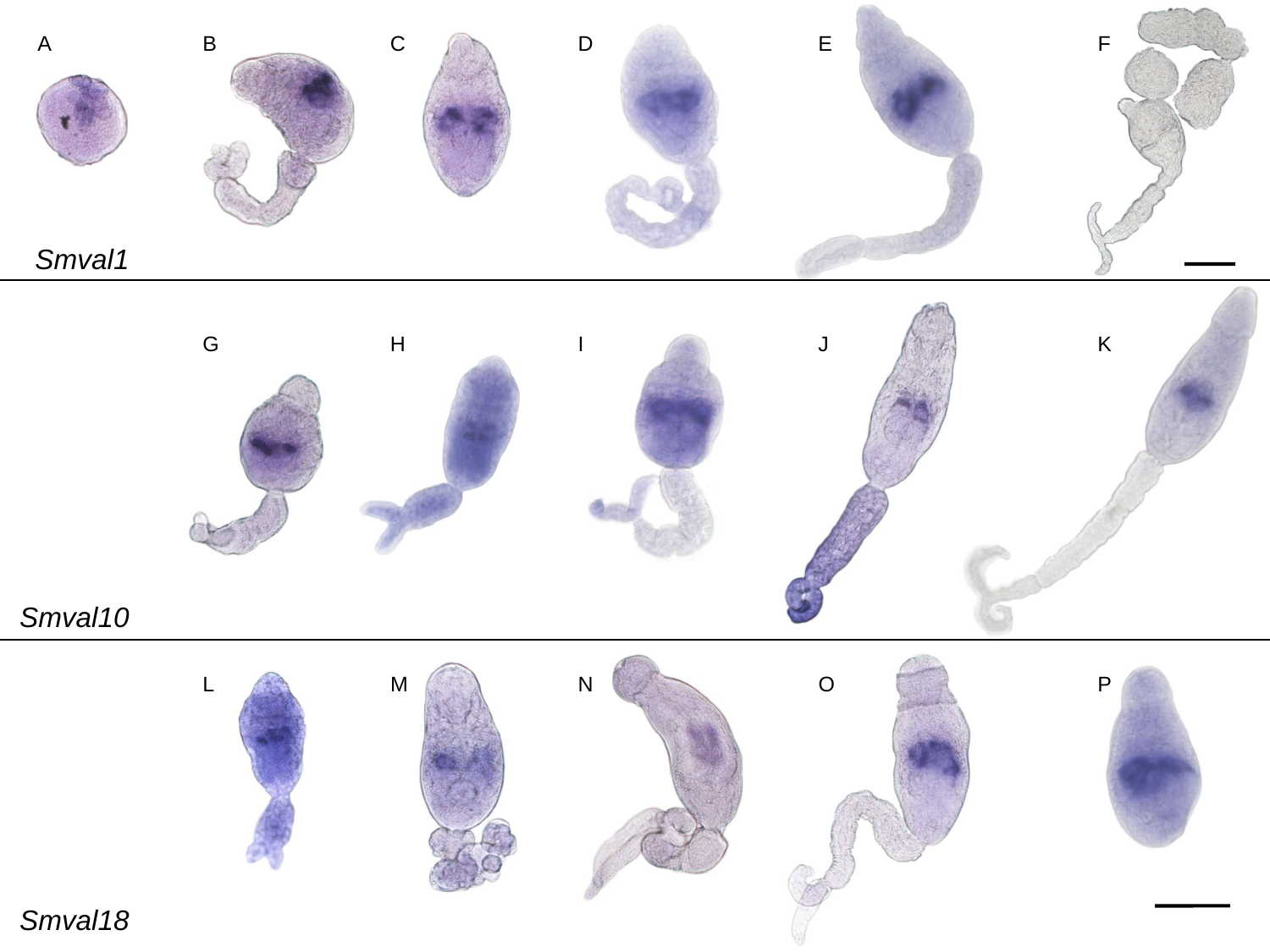

A
B
C
D
E
F
Smval1
G
H
I
J
K
Smval10
L
M
N
O
P
Smval18

Supplement: Supplementary Fig. S4 — Transcript localization of Smvals 1, 10 and 18 in the pre-acetabular glands of germ balls and immature cercariae by Whole in-situ hybridization (WISH). (A) No clear staining was detected in young round germ balls. Transcript expression was identified in the pre-acetabular glands of stubby-tailed developing germ balls (B, G, H and L), as well as in the young elongating-tail stage (C, D, I, J, M, N and O) and in germ balls nearing maturity (E, K and P). (F) Negative control hybridized with sense probe of SmVAL4. Scale bars = 50 μm. [file mmc6.pptx]
